# Correlation of Serum Sodium and Potassium With Thiamin Levels in Type 1 and Type 2 Diabetic Patients

**DOI:** 10.7759/cureus.59416

**Published:** 2024-04-30

**Authors:** Syed Shahiq Ali, Qurat Ul Ain Ismail, Anusha Yusuf, Syed Tariq Ali Adnan, FNU Durga, FNU Priyanka, Hafiza Ayesha Ghersheen, Mehak Ahuja, Adnan Anwar, Atif A Hashmi

**Affiliations:** 1 Internal Medicine, Jinnah Postgraduate Medical Centre, Karachi, PAK; 2 Internal Medicine, Jinnah Medical and Dental College, Karachi, PAK; 3 Emergency Medicine, Supporting Health and Education, Deserving Fellows (SHED) Hospital, Karachi, PAK; 4 Community Medicine, Karachi Medical and Dental College, Karachi, PAK; 5 Internal Medicine, Jinnah Sindh Medical University, Karachi, PAK; 6 Internal Medicine, Bolan Medical College, Quetta, PAK; 7 Internal Medicine, Dow University of Health Sciences, Karachi, PAK; 8 Physiology, Hamdard College of Medicine and Dentistry, Karachi, PAK; 9 Internal Medicine, Anabiya General Hospital, Karachi, PAK; 10 Pathology, Liaquat National Hospital and Medical College, Karachi, PAK

**Keywords:** thiamin, type 2 diabetes mellitus, type 1 diabetes mellitus, serum potassium, serum sodium

## Abstract

Introduction

Chronic metabolic disorders such as diabetes mellitus (DM) are becoming a global health concern. According to recent studies, the pathophysiology of DM may involve factors other than traditional glycemic control, such as electrolyte balance and thiamin status. Therefore, this study evaluated the relationship between sodium and potassium and serum thiamin levels in patients with type 1 and type 2 DM.

Methods

This study was conducted in multiple diabetic outpatient clinics and centers in Karachi, Pakistan, using a non-probability convenience sampling method. The study lasted for approximately six months after the synopsis was approved. A total of 64 patients were selected, 32 of whom each had type 1 and type 2 DM. All patients who were between the ages of 25 and 46 years old and had either type 1 or type 2 DM were included in the study. A Mann-Whitney test and an independent t-test were used to compare the means between the two study groups. Pearson’s correlation and chi-square tests were used to determine the variables, correlations, and associations with type 1 and type 2 DM.

Results

The study findings showed that the distribution of gender among diabetic patients revealed that among males, eight (25.0%) had type 1 DM, and 10 (31.2%) had type 2 DM. Among females, 24 (75.0%) had type 1 DM, and 22 (68.8%) had type 2 DM. Significant correlations were observed in the means of blood glucose levels, such as glycated hemoglobin (HbA1c), fasting blood sugar (FBS), and serum thiamin levels, among patients with type 1 and type 2 DM (p < 0.001). The HbA1c, FBS, and serum thiamin levels were significantly higher in type 2 DM patients than in type 1 DM patients. Among patients with type 1 DM, sodium levels were not substantially correlated with thiamin levels (p = 0.570, r = 0.104), whereas potassium levels were significantly correlated with thiamin levels (p = 0.005, r = 0.263).

Conclusion

We conclude that the sodium level was not significantly correlated with serum thiamin status in type 1 and type 2 DM, whereas a low positive correlation was observed between potassium and serum thiamin levels in type 1 DM. However, there was no significant correlation concerning potassium levels in type 2 DM.

## Introduction

Thiamin is a vital water-soluble vitamin that is required for mitochondrial energy in the synthesis of adenosine triphosphate (ATP). It is a fundamental vitamin that acts as a rate-restricting cofactor for several enzymes used in the energy metabolic reaction. Certain enzymes function at important junctions and entrances in the routes for amino acids, fatty acids, and glucose [1,2]. The dynamic form of free thiamin, thiamin diphosphate (TDP), also known as thiamin pyrophosphate (TPP), is produced by phosphorylating it once it has been absorbed [1,2]. Thiamin has an extremely short half-life and a small space for preservation. Moreover, various substances, such as pharmaceuticals and environmental pollutants, can degrade and deplete it [2]. A diet high in thiamin includes foods like pork, fish, various seeds and nuts, peas, beans, wheat, brown rice, acorn squash, and asparagus [2]. For adult males and females, the recommended daily allowance (RDA) for thiamin is 1.2 mg and 1.1 mg, respectively. The prevalence of thiamin insufficiency in a variety of patient populations (between 20% and more than 90%) has raised concerns that the existing RDA may not be adequate to fulfill the requirements of contemporary life [2].

Diabetes mellitus (DM) is a serious health issue, and its prevalence is rising promptly in all age groups. The three main causes of DM are genetic, environmental, and host factors. The main mechanisms through which diabetes can develop are the metabolic and autoimmune routes. Obesity, a lack of activity, disturbances in hormones, and low absorbency are the primary factors associated with DM. Diabetes mellitus is related to shock, kidney dysfunction, and stroke. The optimal defenses against diabetes, especially type 2 DM, are healthy eating habits and regular physical activity [3,4]. Insulin-dependent type 1 and insulin-independent type 2 DM are the leading categories of DM. Type 2 DM occurs more frequently through the progression of resistance to insulin and reduced sensitivity to the insulin receptor. Type 1 DM arises through an autoimmune attack of pancreatic beta cells and is triggered by the inability of pancreatic beta cells to form insulin [3]. Pakistan and other emerging nations have higher rates of type 2 DM [4]. It has been recognized that reduced thiamin reserves are related to diabetes because thiamin directly affects the metabolism of carbohydrates. Research has shown that patients with type 1 and type 2 diabetes have increased renal clearance of thiamin [5].

Globally, the prevalence of DM has increased significantly in recent decades, which has had a significant impact on economic expenses, health care burden, and quality of life [6]. The International Diabetes Federation (IDF) Diabetes Atlas estimations report that 537 million people globally had DM in 2021, and by 2045, that number is expected to increase to 783 million [7]. Diabetes-related macrovascular problems like stroke or myocardial infarction and microvascular consequences like impaired vision, renal impairment, or amputations in diabetics are linked to significant morbidity and higher mortality rates [8]. One key factor in the pathophysiology of DM is oxidative stress, which redirects upstream metabolites from glycolysis into processes of glucose overuse [9]. An insufficiency of thiamin, a necessary cofactor for multiple enzymes that regulate glycolysis and the Krebs cycle, could be the cause of the excess buildup of these metabolites, indicating the potential involvement of this vitamin in the occurrence of microvascular problems associated with DM [1]. Clinical signs of thiamin deficiency include cardiovascular disorders (CVD) or neurological disorders.

Animal models and a few clinical trials have demonstrated the ability of thiamin and its derivative, benfotiamine, to prevent diabetic microangiopathy [1,10,11]. In vertebrates, thiamin also regulates neuronal and neuromuscular transmission. Severe nervous system illnesses such as Wernicke-Korsakoff syndrome and beriberi may result from thiamin deficiency [1]. Although 80% of the thiamin in the body is in the physiologically active form, TDP concentrations in whole blood are believed to be a helpful indicator of thiamin status [12]. It is possible to remove or add phosphates from the molecule to create thiamin monophosphate (TMP) or thiamin triphosphate (TTP), respectively. However, the benefits of monitoring blood TTP and TMP levels are not immediately apparent [2,12]. Thiamin status is commonly assessed in laboratories by measuring the levels of thiamin, TMP, TDP, TTP, and erythrocyte transketolase in plasma or blood. Diabetics and controls had these indicators evaluated, but the outcomes were not all that similar. For instance, some studies [5,13,14], but not all of them [15-17], found that people with DM had significantly lower amounts of one or more thiamin indicators than people without the disease.

Although thiamin, or vitamin B1, is a necessary component in the metabolism of glucose, it is unknown whether people with DM have lower thiamin levels than those with a normal glucose metabolism. Therefore, this study assessed the correlation between serum thiamin level and serum sodium and potassium in patients with type 1 and type 2 DM.

## Materials and methods

Patient selection

This was a prospective observational cross-sectional study that involved multiple diabetic outpatient centers in Karachi, Pakistan, using a non-probability convenience sampling method. The study duration was six months, from October 2023 to March 2024. This study was approved by the Institutional Review Board of Anabiya General Hospital (Anabiya/23/056). Patients who visited diabetic clinics for disease management were included in the study. Informed written consent was obtained, and the associated benefits and risks were explained to all participants. Patient confidentiality was maintained by removing patient-identifying information from the proforma. A total of 64 patients were selected, of whom 32 each had type 1 and type 2 DM and were aged between 25 and 46. Conversely, diuretic users, people with serious concomitant medical illnesses such as renal impairment, CVD, chronic liver disease, or people who underwent transplant surgery were not included in the study.

Clinical and biochemical parameters

Demographic factors, including age, gender, and comorbidities, were documented. Samples for serum analysis were obtained in heparinized tubes. Blood samples were immediately centrifuged for 20 minutes at 2000 rpm in a non-heparinized tube. Clear supernatant serum was used to assess biochemical diagnostic parameters such as potassium, sodium, fasting lipid profile, random blood sugar (RBS), and thiamin levels. The right arm’s blood pressure was measured twice when the patient was sitting and while standing. For each person, an average of two evaluations from measurements taken five minutes apart were recorded.

Data analysis

Data were entered and analyzed using SPSS Statistics version 26.0 (IBM Corp., Armonk, NY, USA). Descriptive statistics were expressed as means and standard deviations, while gender and coexisting illnesses were documented as percentages and frequencies. The Mann-Whitney test and independent t-test were used to compare the means between patients with type 1 and type 2 DM. The chi-square test and Pearson’s correlation were used to find the associations and correlations between the variables and type 1 and type 2 DM. A p-value of 0.05 was considered significant.

## Results

Demographic details of the population under study

A total of 64 diabetic patients, i.e., 32 patients each in the type 1 DM and type 2 DM groups, were included in the study. The distribution of gender revealed that among males, eight (25.0%) had type 1 DM, and 10 (31.2%) had type 2 DM. Among females, 24 (75.0%) had type 1 DM, and 22 (68.8%) had type 2 DM. The difference in DM types between genders was statistically insignificant (p = 0.578). Hypertension was present in 11 (34.4%) patients with type 1 DM and 20 (62.5%) with type 2 DM. Conversely, among individuals without hypertension, 21 (65.6%) had type 1 DM and 12 (37.5%) had type 2 DM. The association between hypertension and DM type was statistically significant (p = 0.024). In the type 1 DM group, two (6.2%) had neuropathy, whereas there were eight (25.0%) with neuropathy in the type 2 DM group. Among those without neuropathy, 30 (93.8%) had type 1 DM, and 24 (75.0%) had type 2 DM, with a statistically significant difference between neuropathy and diabetes type (p = 0.039). Additionally, a significant relationship was observed between coronary artery disease and both types of diabetes (p = 0.010), as shown in Table 1.

**Table 1:** Demographic details of type 1 and type 2 DM patients (n = 64) *A p-value <0.05 is considered significant. The data has been presented as number (n) and percentage (%). DM: Diabetes mellitus

Demographic details		Study group	n	%	p-value
Gender	Male	Type 1 DM	8	25.0	0.578
		Type 2 DM	10	31.2	
	Female	Type 1 DM	24	75.0	
		Type 2 DM	22	68.8	
Hypertension	Yes	Type 1 DM	11	34.4	0.024*
	No	Type 1 DM	21	65.6	
	Yes	Type 2 DM	20	62.5	
	No	Type 2 DM	12	37.5	
Neuropathy	Yes	Type 1 DM	2	6.2	0.039*
	No	Type 1 DM	30	93.8	
	Yes	Type 2 DM	8	25.0	
	No	Type 2 DM	24	75.0	
Coronary artery disease	Yes	Type 1 DM	2	6.2	0.010*
	No	Type 1 DM	30	93.8	
	Yes	Type 2 DM	10	31.2	
	No	Type 2 DM	22	68.8	

Differences in the clinical parameters of type 1 and type 2 DM patients

The association of demographics among type 1 and type 2 DM showed statistically significant variations in all means of the main demographics, such as age (p < 0.001), BMI (p < 0.001), and duration of DM (p < 0.001). The age, BMI, and disease duration were higher in type 2 DM than in type 1 DM. In contrast, statistically insignificant differences were observed among patients with type 1 and 2 DM concerning systolic and diastolic blood pressure (p = 0.065 and 0.260, respectively), as shown in Table 2.

**Table 2:** The association of demographics and vitals among type 1 and 2 DM patients *A p-value <0.05 is considered significant. The data has been presented as mean and standard deviation. DM: Diabetes mellitus

Variables	Study group	Mean	Standard deviation	p-value
Age (years)	Type 1 DM	25.06	7.07	<0.001*
	Type 2 DM	42.87	10.26	
Body mass index (kg/m^2^)	Type 1 DM	17.54	8.04	<0.001*
	Type 2 DM	32.29	5.73	
Duration of DM (years)	Type 1 DM	4.06	2.03	<0.001*
	Type 2 DM	9.09	2.78	
Blood pressure systolic (mm Hg)	Type 1 DM	128.44	13.7	0.065
	Type 2 DM	135.0	13.4	
Blood pressure diastolic (mm Hg)	Type 1 DM	88.75	13.61	0.260
	Type 2 DM	91.56	11.1	
Heart rate (beats per minute)	Type 1 DM	77.03	8.49	0.227
	Type 2 DM	79.25	5.76	

Comparison of biochemical parameters of type 1 and type 2 DM patients

Statistically significant differences were observed between patients with type 1 and type 2 DM in terms of fasting blood sugar (FBS), glycated hemoglobin (HbA1c), and serum thiamin levels (p < 0.001). The HbA1c, FBS, and serum thiamin levels were significantly higher in type 2 DM than in type 1 DM. In contrast, insignificant differences were observed among patients with type 1 and type 2 DM for RBS (p = 0.196). The mean blood sodium level had a significant association between type 1 and type 2 DM (133.6 ± 2.35 vs. 135.5 ± 2.32, p = 0.002). Additionally, the mean blood potassium level had an insignificant association between type 1 and type 2 DM (4.05 ± 0.30 vs. 4.19 ± 0.30, p = 0.098), as presented in Table 3.

**Table 3:** The association of blood glucose levels, electrolytes, and levels of thiamin among type 1 and 2 DM patients *A p-value <0.05 is considered significant. The data has been presented as mean and standard deviation. DM: Diabetes mellitus; HbA1c: Glycated hemoglobin

Variables	Study group	Mean	Standard deviation	p-value
HbA1c (%)	Type 1 DM	7.33	0.88	< 0.001*
	Type 2 DM	9.38	1.91	
Fasting blood sugar (mg/dl)	Type 1 DM	147.5	47.01	< 0.001*
	Type 2 DM	211.03	70.0	
Random blood sugar (mg/dl)	Type 1 DM	258.7	51.8	0.196
	Type 2 DM	279.2	46.5	
Serum thiamine	Type 1 DM	7.28	1.86	< 0.001*
	Type 2 DM	15.05	4.71	
Sodium (mEq/l)	Type 1 DM	133.6	2.35	0.002*
	Type 2 DM	135.5	2.32	
Potassium (mEq/l))	Type 1 DM	4.05	0.30	0.098
	Type 2 DM	4.19	0.30	

Correlation of serum thiamin with serum electrolytes in type 1 and type 2 diabetic patients 

Among patients with type 1 DM, the sodium level was insignificantly correlated with serum thiamin status (r = 0.104, p = 0.570), whereas the potassium level was significantly correlated (low positive correlation) with serum thiamin status (r = 0.263, p = 0.005). Similarly, an insignificant correlation was observed between the sodium level and serum thiamin status (r = 0.488, p = 0.640) in type 2 DM, and the potassium level was insignificantly correlated with serum thiamin status (r = -0.097, p = 0.598), as presented in Table 4.

**Table 4:** The correlation of sodium and potassium levels with thiamine status in type 1 and 2 DM patients *A p-value <0.05 is considered significant. DM: Diabetes mellitus; r: Pearson correlation coefficient

Groups	Variables	Serum thiamin status	
		r	p-value
Type 1 DM (n=32)	Sodium	0.104	0.570
	Potassium	0.263	0.005*
Type 2 DM (n=32)	Sodium	0.488	0.640
	Potassium	- 0.097	0.598

## Discussion

In this study, we found a low positive correlation between serum potassium and thiamin levels in type 1 DM patients. Low thiamin reserves have been linked to diabetes because thiamin affects the body’s ability to metabolize carbohydrates. Research has shown that patients with type 1 and type 2 DM have increased renal clearance of thiamin [18]. Thus, this study demonstrates an association between sodium and potassium levels and thiamin status in patients with type 1 and type 2 DM.

This cross-sectional study was performed at multiple diabetic centers in Karachi, Pakistan. The study findings showed substantially significant changes (p < 0.001) in the mean blood glucose levels, including HbA1c, FBS, RBS, and thiamin levels, between patients with type 1 and type 2 DM and controls. In addition, it was demonstrated that the thiamin levels of type 1 and type 2 DM patients and the control group’s HbA1c and FBS were not significantly correlated, whereas the combined diabetic groups’ HbA1c and FBS had a significant correlation with the thiamin level [19]. The present study was partially consistent with the abovementioned study and revealed a statistically significant relationship between type 1 and type 2 DM in terms of blood sugar levels such as HbA1c, FBS, and serum thiamin (p < 0.001), except for RBS, which was insignificantly associated with type 1 and type 2 DM. As far as the correlation is concerned, potassium level had a significant correlation with serum thiamin status in type 1 diabetes (r = 0.263, p = 0.005).

Similarly, a different study revealed that serum thiamin levels were lower in patients with type 1 DM than in those with type 2 DM and their healthy counterparts [20]. In a recent investigation, thiamin levels were substantially lower in patients with type 1 DM compared with the control group [21]. The present study supported the above studies and showed that thiamin levels were significantly lower in type 1 DM patients (7.28 ± 1.86) than in type 2 (15.05 ± 4.71) patients (p < 0.001).

Remarkably, another study revealed an inverse relationship between blood glucose levels and thiamin levels, with the level of thiamin in the blood of patients with type 1 DM being considerably lower than that in healthy controls [2]. Another study found that thiamin levels in the blood were significantly reduced in 18 (60%) type 1 DM patients compared with healthy controls [22]. The present study demonstrates that patients with type 1 DM had significantly lower thiamin levels than those with type 2 DM (p < 0.001), which was in agreement with previously published outcomes.

Interestingly, another comparative study evaluated thiamin levels in patients with type 1 and 2 DM. They found that these patients had a significant increase in RBS, FBS, and HbA1c levels compared with the control group. This study’s interesting finding, which agrees with another study, was that patients with type 1 and type 2 DM had considerably lower mean thiamin levels than healthy individuals [23]. Another study conducted in 2015 found that people with type 1 DM had significantly higher glucose levels than controls (p = 0.001) [22]. Similarly, studies have shown that patients with type 1 and type 2 DM have significantly higher HbA1c levels than controls [18]. In a 2003 study, HbA1c values were greater in patients with DM than in healthy individuals (p =0.002). Furthermore, HbA1c has been suggested as a valuable and accurate way to screen for and diagnose DM [23]. These results support the present study and reveal that patients with type 2 DM had significantly elevated FBS, HbA1c, and thiamin levels compared to patients with type 1 DM (p < 0.001).

Likewise, a comparative analysis of hematological and biochemical markers in patients with diabetes was performed at many diabetic centers in Karachi. According to the study findings, patients with type 2 DM had mean FBS, RBS, and HbA1c levels that were considerably higher than those of either type 1 DM or the controls (p < 0.05). Additionally, it was also revealed that individuals with type 1 or type 2 DM had considerably lower thiamin levels than controls (14.89 ± 4.82 and 7.35 ± 1.90 vs. 69.56 ± 12.75, p < 0.001) [18]. According to an additional study, patients with type 1 diabetes had substantially greater glucose levels than healthy individuals (p = 0.001) [22]. It was also shown that HbA1c levels were considerably higher in patients with type 1 and type 2 DM than in controls. Likewise, another study also found that diabetic patients had greater HbA1c levels than non-diabetic individuals (p = 0.002). Furthermore, it has been recommended that HbA1c is a very useful and specific screening and investigative tool for diabetes [23]. In the present study, patients with type 2 DM had considerably greater mean FBS and HbA1c levels than those with type I DM (p < 0.001). Concerning thiamin levels, it was observed that significantly higher levels were found in type 2 DM patients than in type 1 DM patients (15.05 ± 4.71 vs. 7.28 ± 1.86, p < 0.001).

According to another study, type 1 DM patients had significantly reduced thiamin levels compared with controls (p = 0.002) [21]. A study performed in 2007 also discovered a substantial decrease in plasma thiamin levels (p < 0.001) between patients with type 1 and type 2 DM and normal controls [24]. Similarly, a 2012 study found that patients with type 1 and type 2 DM had significantly lower blood thiamin levels than controls (p < 0.001) [5]. The present study was not in agreement with the above-reported studies and showed that thiamin levels were significantly lower in type 1 DM than in type 2 DM (p < 0.001).

Limitations

This study has certain limitations. This study may have had selection bias because of observer bias and a non-probability sampling method. To obtain more precise results, future research that employs the probability sampling method should investigate this association with larger sample sizes. Moreover, because this was a multicenter trial, the sample size was small. Due to the limited sample size, the findings cannot be generalized to the whole population. Therefore, we recommend that large sample-sized studies be conducted to evaluate the correlation between serum thiamin and electrolytes. Furthermore, this study did not conduct a long-term follow-up to observe the relationship between serum thiamin levels and diabetic complications.

## Conclusions

This study concludes that sodium level is insignificantly correlated with serum thiamin status in type 1 and type 2 DM and that a low positive correlation is observed between potassium level and serum thiamin level in type 1 DM. However, there was an insignificant correlation concerning potassium level in type 2 DM. Further research is warranted to elucidate the mechanistic details and establish clinical guidelines for optimizing electrolyte balance and thiamin status in individuals with DM. Large-scale studies are needed to establish this correlation.

## References

[1] Beltramo E, Mazzeo A, Porta M (2021). Thiamine and diabetes: back to the future?. Acta Diabetol.

[2] Marrs C, Lonsdale D (2021). Hiding in plain sight: modern thiamine deficiency. Cells.

[3] American Diabetes Association (2005). Standards of medical care in diabetes. Diabetes Care.

[4] American Diabetes Association (2014). Diagnosis and classification of diabetes mellitus. Diabetes Care.

[5] Al-Attas OS, Al-Daghri NM, Alfadda AA, Abd-Alrahman SH, Sabico S (2012). Blood thiamine and its phosphate esters as measured by high-performance liquid chromatography: levels and associations in diabetes mellitus patients with varying degrees of microalbuminuria. J Endocrinol Invest.

[6] Harding JL, Pavkov ME, Magliano DJ, Shaw JE, Gregg EW (2019). Global trends in diabetes complications: a review of current evidence. Diabetologia.

[7] Magliano DJ, Boyko EJ, IDF Diabetes Atlas Scientific Committee (2021). IDF Diabetes Atlas. Brussels: International Diabetes Federation.

[8] Gregg EW, Li Y, Wang J (2014). Changes in diabetes-related complications in the United States, 1990-2010. N Engl J Med.

[9] Brownlee M (2001). Biochemistry and molecular cell biology of diabetic complications. Nature.

[10] Hammes HP, Du X, Edelstein D (2003). Benfotiamine blocks three major pathways of hyperglycemic damage and prevents experimental diabetic retinopathy. Nat Med.

[11] Ziegler D, Porta M, Papanas N (2022). The role of biofactors in diabetic microvascular complications. Curr Diabetes Rev.

[12] Whitfield KC, Bourassa MW, Adamolekun B (2018). Thiamine deficiency disorders: diagnosis, prevalence, and a roadmap for global control programs. Ann N Y Acad Sci.

[13] Al-Daghri NM, Al-Attas OS, Alkharfy KM, Alokail MS, Abd-Alrahman SH, Sabico S (2013). Thiamine and its phosphate esters in relation to cardiometabolic risk factors in Saudi Arabs. Eur J Med Res.

[14] Waheed P, Naveed AK, Ahmed T (2013). Thiamine deficiency and its correlation with dyslipidaemia in diabetics with microalbuminuria. J Pak Med Assoc.

[15] Chalásová K, Pácal L, Pleskačová A (2018). Transketolase activity but not thiamine membrane transport change in response to hyperglycaemia and kidney dysfunction. Exp Clin Endocrinol Diabetes.

[16] Nix WA, Zirwes R, Bangert V, Kaiser RP, Schilling M, Hostalek U, Obeid R (2015). Vitamin B status in patients with type 2 diabetes mellitus with and without incipient nephropathy. Diabetes Res Clin Pract.

[17] Polizzi FC, Andican G, Çetin E, Civelek S, Yumuk V, Burçak G (2012). Increased DNA-glycation in type 2 diabetic patients: the effect of thiamine and pyridoxine therapy. Exp Clin Endocrinol Diabetes.

[18] Anwar A, Azmi MA, Siddiqui JA, Panhwar G, Shaikh F, Ariff M (2020). Thiamine level in type I and type II diabetes mellitus patients: a comparative study focusing on hematological and biochemical evaluations. Cureus.

[19] Khan MU, Mubeen M, Chohan HK (2023). Correlation of fasting blood sugar and glycated hemoglobin (HbA1c) with thiamine levels in diabetic patients. Cureus.

[20] Raza L, Raza U, Adil R (2022). Association of age, gender and type of diabetes with thiamine level: a cross-sectional analysis. J Pharm Res Int.

[21] Tai VML (2016). A case report on the use of oral thiamine in a palliative care patient in the management of peripheral edema in a community setting in New South Wales, Australia. Int J Case Rep Images.

[22] Al-Daghri NM, Alharbi M, Wani K, Abd-Alrahman SH, Sheshah E, Alokail MS (2015). Biochemical changes correlated with blood thiamine and its phosphate esters levels in patients with diabetes type 1 (DMT1). Int J Clin Exp Pathol.

[23] Greci LS, Kailasam M, Malkani S, Katz DL, Hulinsky I, Ahmadi R, Nawaz H (2003). Utility of HbA(1c) levels for diabetes case finding in hospitalized patients with hyperglycemia. Diabetes Care.

[24] Thornalley PJ, Babaei-Jadidi R, Al Ali H (2007). High prevalence of low plasma thiamine concentration in diabetes linked to a marker of vascular disease. Diabetologia.

